# Urinothorax and Empyema: Presentation of an Unusual Complication of a Rare Condition

**DOI:** 10.1155/crpu/5592208

**Published:** 2025-12-17

**Authors:** Nolan Fox, Nicholas Fox, Shourjo Chakravorty, Neeraj Sinha

**Affiliations:** ^1^ Internal Medicine Residency Program, Johns Hopkins Bayview Medical Center, Baltimore, Maryland, USA, hopkinsbayview.org; ^2^ Internal Medicine Residency Program, Temple University Hospital, Philadelphia, Pennsylvania, USA, temple.edu; ^3^ Division of Pulmonary, Allergy and Critical Care, Thomas Jefferson University Hospital, Philadelphia, Pennsylvania, USA, jefferson.edu; ^4^ Lung Transplantation Program, Thomas Jefferson University Hospital, Philadelphia, Pennsylvania, USA, jefferson.edu

## Abstract

A 68‐year‐old male with a history of metastatic bladder cancer and urinary obstruction presented with intractable hiccups and chest pain is presented in this case. He was found to have a unilateral pleural effusion in addition to ipsilateral renal metastasis abutting the diaphragm. Imaging revealed empyema. Thoracentesis revealed positive cultures and a lack of malignant cells on cytology. Comorbid urinary obstruction and urinary tract infection suggested the rare diagnosis of an infected urinothorax. Thoracentesis also revealed pleural fluid creatinine greater than serum creatinine, meeting the most specific diagnostic criteria for urinothorax and establishing the diagnosis. Treatment aimed at evacuating the pleural space without addressing the comorbid bladder obstruction led to an unfavorable outcome in this case.

## 1. Introduction

Urinothorax (UT) is a rare and underreported cause of pleural effusion, which occurs when urine collects in the pleural space [1, 2]. UT is commonly seen as a complication of surgical procedures involving the kidney and urinary system, but can also occur secondary to urinary obstruction, trauma, or malignant disease [1–3]. UT is often unilateral and shows a predominance in men. It is often undiagnosed because of a lack of symptoms, but can, in some cases, manifest with significant dyspnea and hypoxemia [2].

Infection can occur in the pleural space in the setting of UT. A previous review found that 9.3% of UT cases returned with positive cultures [4]. The progression of an infected UT to a multiloculated empyema can also occur with significant associated morbidity [5]. Here, we present a case of UT occurring secondary to obstructive nephropathy and progressing to empyema.

## 2. Case Presentation

A 68‐year‐old male with metastatic bladder cancer, stage five chronic kidney disease, and previous pulmonary embolism presented with intractable hiccups and chest pain is presented in this case. He was first diagnosed with invasive urothelial carcinoma 10 years prior; at which time he underwent cyst prostatectomy with creation of an ileal conduit. His bladder cancer later recurred, and he underwent bilateral diversionary ureteral stent placement to treat obstructive uropathy. Four years ago, he was found to have urothelial carcinoma metastasis to the left kidney confirmed by biopsy. At that time, he refused nephrectomy and instead began treatment with the immunotherapy Padcev (Enfortumab‐Vedotin). He was then lost to follow‐up for several years. In the month leading up to this admission, he presented to the emergency department multiple times with chest pain, shortness of breath, and hiccups. During these visits, he was found to have a large left simple pleural effusion compressing his lung parenchyma.

On the present admission, he was reporting intractable hiccups, left‐sided chest pain and shortness of breath. He was initially afebrile and tachycardic to the 130 s with stable blood pressure. Shortly after admission, he began spiking fevers as high as 39.4°C. He maintained a blood oxygen saturation of greater than 95% on room air. Exam revealed a thin, ill‐appearing male with scleral icterus and absent left‐sided breath sounds.

**Figure 1:**
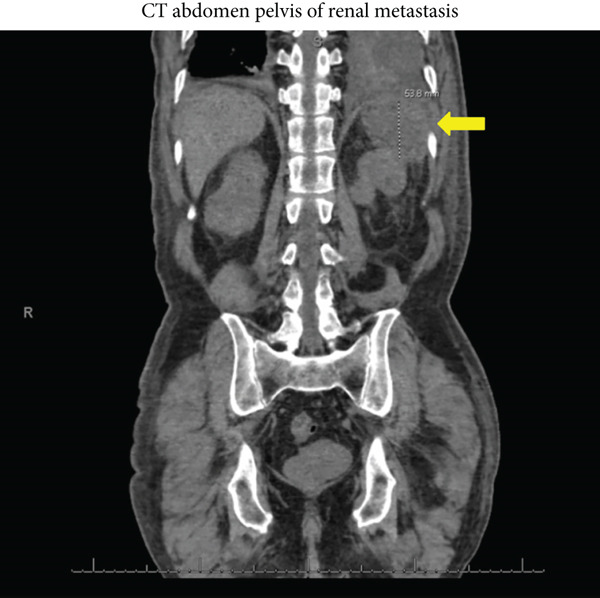
A CT abdomen pelvis in the sagittal view showing the patient’s left‐sided renal metastasis (yellow arrow) abutting the diaphragm.

**Figure 2:**
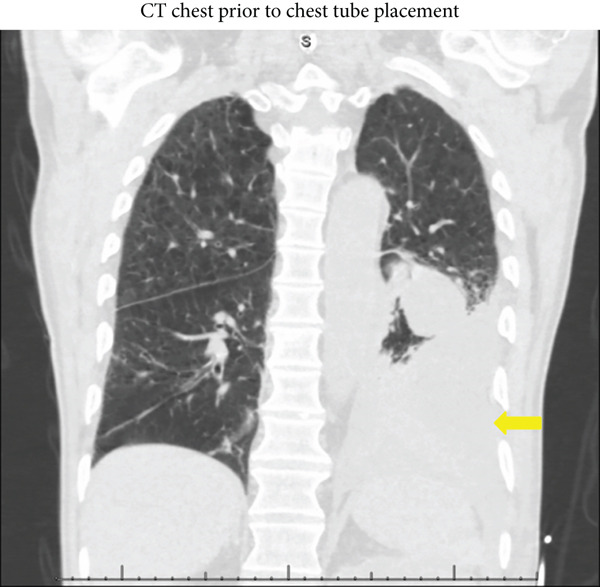
A CT chest in the coronal view taken prior to chest tube placement displaying a large loculated collection (yellow arrow) throughout the left lung base.

His white blood cell count was 26,200 cells/*μ*L with a neutrophilic predominance. CT imaging revealed a mass in the left renal pelvis abutting the left diaphragm (Figure 1), as well as a large heterogeneous collection in his left lung concerning for an empyema (Figure 2). A bedside thoracentesis removed 550 mL of exudative pleural fluid with the following properties: white blood cell count of 37,400 cells/*μ*L, glucose less than 2 mg/dL, pH of 7.11, and LDH of 2,281 IU/L. Pleural fluid protein was 4.9 mg/dL, while serum protein level was 6.0 mg/dL. Pleural fluid creatinine was 4.29 mg/dL while serum creatinine was 4.11 mg/dL. Culture of the pleural fluid grew *Staphylococcus constellatus* and *Bacteroides thetaiotaomicron*. Urine culture growth was polymicrobial with multiple gram‐positive and gram‐negative organisms.

Broad spectrum empiric antibiotics were initiated shortly after admission, which were narrowed to ceftriaxone and metronidazole once pleural fluid culture results returned. Given the size and loculation of the effusion, and the presence of persistent fevers, it was deemed necessary to provide an additional intervention for source control. Because of his frailty, thoracic surgery intervention was deferred, and a chest tube was placed in the left basilar segment of his pleural space. Within the first 48 h, the tube drained nearly 2 L of purulent material. When output slowed, the tube was flushed with a dornase solution. TPA was not given out of concern that his malignancy and malnourished status significantly increased his risk of hemorrhage. This compromise led to additional output over several days.

**Figure 3:**
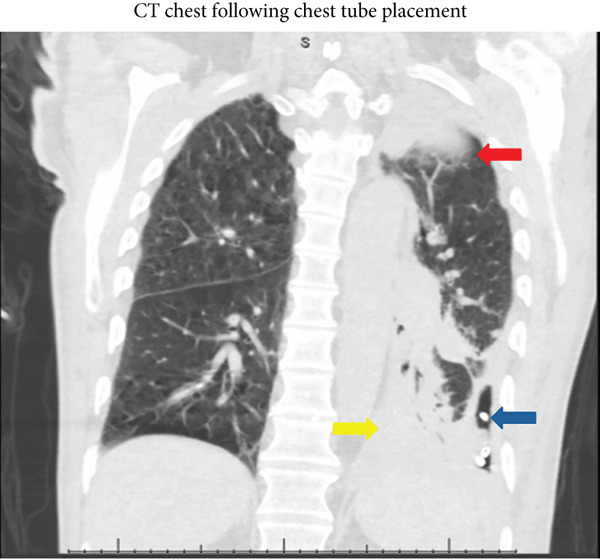
A CT chest in the coronal view after chest tube placement and drainage displaying a reduced basilar collection (yellow arrow) with pigtail catheter in place (blue arrow) and a persistent apical collection (red arrow).

Following placement of the chest tube, the patient reported subjective improvement in his chest pain and ease of breathing. His WBC count began to downtrend from a peak of 27,000 back to his previous outpatient chronically elevated baseline of 20,000 cells/*μ*L. Throughout this time, he remained tachycardic with a max heart rate of 111 bpm and maintained a low‐grade fever peaking at 38.3°C. Radiographically, the basilar component of his effusion decreased moderately in size, but loculated components persisted (Figure 3). He also had a persistent apical collection of loculated effusion, which did not change in response to the chest tube and flushing. Given the extent of his malignancy and apparent inability to clear his infection, the patient and his family were engaged in goals of care discussions. Despite initial desires to continue pursuing curative options, the patient and his family ultimately opted for hospice. Two weeks following placement, the chest tube was removed, and the patient was discharged home on comfort care measures.

## 3. Discussion

This patient was found to have a large, unilateral, exudative pleural effusion with loculations, and a positive culture, consistent with empyema. One plausible mechanism as to how this patient’s effusion developed is that urine accumulated in the peritoneal cavity as a consequence of prior cyst prostatectomy with ileal conduits complicated by outlet obstruction and ureteral stent failure. This build‐up of peritoneal urine then accumulated in the pleural space by way of hydrostatic forces, forming a UT. Any bacteria present in the urinary tract would also be brought over with the urine, leading to an infected pleural effusion.

The patient also had metastatic urothelial carcinoma with a new left kidney metastasis abutting the left diaphragm, suggesting that metastatic invasion of his pleural space could have led to development of a malignant pleural effusion, which eventually became infected. While malignant pleural effusion seemed to be a plausible explanation for a unilateral pleural effusion in a patient with metastatic bladder cancer abutting his left diaphragm, his pleural fluid cytology was negative for malignant cells on two separate samplings and showed only abundant inflammatory cells. It should be noted that pleural fluid cytology has only a modest sensitivity for detecting malignancy, and the infectious process superimposed on this pleural effusion may have further diluted the sensitivity.

The pleural fluid analysis revealed a pleural fluid creatinine greater than his serum creatinine, meeting the primary criteria for UT. His imaging findings revealed obstructive uropathy secondary to metastatic disease, which may lead to UT. His urine was also growing both gram‐positive and gram‐negative bacteria, which correlates with the *Streptococcus* and *Bacteroides* species isolated from his pleural fluid, further supporting the diagnosis of UT. While the patient’s fluid protein value of 4.9 mg/dL was higher than typically expected for UT (under 1.0 mg/dL or less than 50% of serum protein), this value can be inflated in the setting of inflammation or malignancy, meaning clinical suspicion of UT should supersede this diagnostic value [4].

UT is a rare cause of pleural effusions that in this case, was not immediately recognized. While UT most frequently occurs because of trauma, obstructive uropathy is also a documented etiology. Another example of this combination of events was recently described by Glozman et al., which similarly occurred in a patient with a renal mass causing obstructive uropathy that leads to the development of UT [6]. There is a lack of large‐scale data supporting definitive diagnostic criteria for UT. However, pleural creatinine greater than serum creatinine is the most specific diagnostic criteria for UT, with higher ratios correlating with higher specificity. The pH is also often normal or low in UT [4]. Both conditions were met in this case.

The management of UT is not well defined, but literature suggests that treatment aimed at correcting the underlying uropathy is much more effective than thoracentesis or thoracic drainage alone. One review found that outcome was favorable in 96% of patients when treatment was directed towards uropathy with or without thoracic drainage, compared with just over 6% of patients having favorable outcomes when receiving thoracic intervention alone without addressing the underlying uropathy [4]. This underscores the importance of close collaboration with a urologist for managing cases of UT. During this patient’s hospitalization, his acute pulmonary symptoms were prioritized with interventions to evacuate his pleural space and treat his empyema with IV antibiotics. The source of his obstructive uropathy, metastatic urothelial carcinoma, was thought to not be curable or reversible given his advanced disease, and measures were not taken to relieve the obstructive uropathy. In the case described by Glozman et al., ureteral stent placement resulted in resolution of the patient’s acute illness and a favorable outcome [6]. Our patient had extensive metastatic disease, and a favorable outcome was unlikely. The typical combination of intrapleural fibrinolytics (Alteplase + DNaseB) was deferred in favor of DNaseB alone in this case given the patient’s frailty and high bleeding risk. Prior studies have shown variable effects of this combination, and a need for specific monitoring in high risk patients such as this [7]. Pleural irrigation with saline is another treatment option that was considered but deferred given the high risk and low likelihood of improvement given the extent of loculation. The other interventions performed were with the intention of symptoms relief rather than resolution of his empyema. However, interventions aimed at treating the underlying obstructive uropathy rather than the pleural effusion alone may have led to more substantial symptom control or a more favorable outcome.

Although UT remains understudied without definitive treatment guidelines, data does exist supporting the benefit of prioritizing treatment of uropathy when present. More research is needed to further support the proposed diagnostic and treatment options. Increasing the awareness of UT through discussion of cases such as this may promote more effective management of future cases.

## 4. Learning Points

∘ Consider UT in the differential for a pleural effusion in patients with comorbid urinary obstruction or pathology.
∘ Understand and apply the existing diagnostic criteria for UT.
∘ Prioritize treatment of obstructive uropathy in patients with resultant UT to improve patient outcomes.

**Nomenclature**
TPA: tissue plasminogen activator
UT: urinothorax
WBC: white blood cell

## Consent

All the patients allowed personal data processing and informed consent was obtained from all individual participants included in the study.

## Disclosure

The views expressed in this article are of the authors’ possession and not an official position of the institutions they represent.

## Conflicts of Interest

The authors declare no conflicts of interest.

## Funding

No funding was received for this manuscript.

## Data Availability

The data that support the findings of this study are available on request from the corresponding author. The data are not publicly available due to privacy or ethical restrictions.
